# Critical appraisal of diagnostic laboratory tests in the evaluation of central precocious puberty

**DOI:** 10.3389/fped.2024.1504874

**Published:** 2025-01-21

**Authors:** Kanthi Bangalore Krishna, Luigi Garibaldi

**Affiliations:** Division of Pediatric Endocrinology and Diabetes, Department of Pediatrics, UPMC Childrens Hospital of Pittsburgh, University of Pittsburgh, Pittsburgh, PA, United States

**Keywords:** puberty, precocious puberty, GnRHa, LH, FSH

## Abstract

Pubertal onset is characterized by reactivation of the hypothalamic-pituitary-gonadal axis resulting in pulsatile gonadotropin secretion and subsequent sex steroid production. Accurate measurements of the gonadotropins and sex steroids are essential to ensure timely diagnosis of precocious puberty, so as to determine optimal management. This review summarizes the available laboratory testing for the diagnosis of puberty, discussing the different assays used while reviewing the limitations of such testing.

## Introduction

Puberty is the process through which reproductive competence is achieved. Physical characteristics associated with this process include the development of secondary sex characteristics, acceleration in height velocity, and the occurrence of menarche in women and spermatogenesis in men (1). The exact signals that initiate puberty have only recently begun to be understood and several key targets have been identified. Pubertal onset is accompanied by increased kisspeptin and neurokinin B secretion causing the gonadotropin releasing-hormone (GnRH) neurons to secrete GnRH in a pulsatile manner which then stimulates pulsatile pituitary luteinizing hormone (LH) and follicle stimulating hormone (FSH) secretion (2, 3). The LH and FSH further stimulate gonadal sex steroid secretion which promotes development of secondary sex characteristics and influences hypothalamic-pituitary function via negative feedback inhibition (4, 5). In females only, a positive sex steroid feedback develops late in puberty to trigger ovulation (6). Pubertal maturation typically starts between ages 8–13 years in girls and 9–14 years in boys (7). Central precocious puberty (CPP) is associated with early maturation of the hypothalamic-pituitary-gonadal (HPG) axis with premature reactivation of the GnRH pulse generator and sequential maturation of breasts and pubic hair in females and testicular volume, penile enlargement, and pubic hair in males. A significant long-term consequence of untreated CPP is accelerated skeletal maturation, which can result in premature epiphyseal fusion and, consequently a failure to reach genetic target height range (8, 9). Effective CPP treatment could increase final adult height and improve the likelihood of achieving one's genetic target height range (10). Boys with early-onset puberty may have behavioral difficulties and poor psychological adjustment, and girls may experience increased stress from early breast development and onset of menses (11, 12). Therefore, there is a need for accurate diagnosis and identification of children with CPP to optimize treatment plans.

In the evaluation of CPP, in addition to physical examination (for evidence of testicular enlargement in boys and breast development in girls) (13, 14) or ultrasound examination (for evidence of enlarging ovaries/uterus in girls) (15), the most important means of defining the etiology of the precocious sexual development is to determine whether the HPG axis is activated or quiescent by appropriate endocrine tests (16, 17). Those currently available to demonstrate activation of the HPG axis are measurements of one or more of the following: random serum gonadotropin and sex hormone concentrations, urinary gonadotropins, spontaneous nocturnal gonadotropin secretion, and the hormonal (gonadotropin and sex-hormone) response to stimulation with GnRH or a GnRH-agonist (GnRHa). The diagnostic cutoffs of the concentrations of these hormones have varied depending on the assay used and therefore knowledge of the various assays, their sensitivity and specificity are paramount for clinical decision making. These tests are reviewed and their value in diagnosing pubertal onset is appraised below.

## Gonadotropin measurements in the diagnosis of central precocious puberty (CPP): general considerations

Until the 1980's, gonadotropins were quantitated by radioimmunoassay (RIA), a method which allowed reliable measurement of LH and FSH at well detectable (approx. 3–4 IU/L or higher for LH, lower for FSH) serum concentrations, but could not accurately quantitate the low LH levels occurring in the early stages of puberty, due to limited sensitivity and specificity of the assay in the low LH range. This caused an overlap in serum LH concentrations between prepubertal and pubertal children (18, 19). The hormonal diagnosis of puberty required, in most instances, performance of a GnRH stimulation test, in which an LH level of 5 IU/L or greater was usually considered consistent with central puberty. When sensitive and specific immunoradiometric assays (IRMA), based on a “sandwich” assay, employing two antibodies (20, 21) for the subunits of LH, were developed in the mid 80's (22), it became apparent that small nocturnal LH pulses occur in young prepubertal children. There is an increase in frequency and amplitude in the late prepubertal period, so that between late prepuberty and Tanner stage II, LH concentrations become progressively detectable also in random daytime samples, earlier in boys than in girls, as puberty advances (19, 23). Thus, measurements of serum LH concentrations by the new highly sensitive assays allowed, for the first time, the hormonal diagnosis of puberty in random LH samples (19, 24), and appeared to better reflect the LH bioactivity^1^ (26, 27). The immunometric assays for LH evolved in the following years, with transition from the IRMA (functional sensitivity^2^ of about 0.15–0.4 IU/L) to ultrasensitive or highly sensitive immunofluorimetric assays (IFMA) (28) and chemiluminescent (ICMA) assays (29). These new assays (functional sensitivity of 0.02–0.05 IU/L) documented the pulsatile LH secretory activity occurring in the late prepubertal and early pubertal stages accurately, and provided reference data for random LH levels in different pediatric ages and across the pubertal stages for both sexes (23, 29–33). With regard to FSH, the highly sensitive assays showed good correlation with the RIA even at lower concentrations (23), but did not improve the diagnostic yield of measuring random FSH values for the hormonal detection of puberty, due to the substantial overlap of both baseline and GnRH-stimulated levels between prepuberty and early-mid puberty. Conversely, very low FSH concentrations, measurable for the first time by immunometric assays, became a useful marker for the diagnosis of hypogonadotropic hypogonadism (34), which can be confirmed by low FSH, LH response to GnRH stimulation as well (33). We refer the reader to a review article of gonadotropin assays (35).

Although highly sensitive assays are essential to detect the early increase in LH levels at the beginning of normal, precocious or delayed puberty in random samples, most small or non-pediatric hospitals in the US, employ LH assays (such as automated ICMAs) on laboratory “platforms” used to test several other analytes. While these assays have sufficient clinical sensitivity (limit of detection 0.1 IU/L, functional sensitivity usually ∼0.2 IU/L) (36, 37) to measure LH in children with mid-advanced puberty, and good specificity, they are often inadequate to detect the low LH concentrations occurring at the onset of puberty. Of note, assays of different sensitivity may be produced by the same manufacturer [for example, HS-DELFIA with an LH functional sensitivity of 0.05 IU/L (38), vs. autoDELFIA (39), with sensitivity of 0.6 IU/L] making it important to focus on the sensitivity of the assay quoted in different papers.

The other critical issue, which is rarely discussed about these assays is that of (excessive) specificity. While the monoclonal antibodies currently employed in most LH immunometric assays have been useful in assuring reproducibility of the assays over time and minimal cross reactivity with beta-hCG, the possibility of “excessive specificity” should be kept in mind as a factor that may lead to “inappropriately” low LH levels. Unlike other hormones secreted in a definite molecular form (such as T3 and T4), the glycoprotein hormones, including LH, circulate in different isoforms, related to post-translational modifications in glycosylation, sialylation and sulfation affecting the mass and content of the carbohydrate moiety, as well as the charge of the molecule. While changes in the carbohydrate component do not interact directly with the antibodies to LH, which are directed against the amino acid/protein epitopes, they may indirectly affect the binding of LH to the Ab in the immunoassay and to its receptor, resulting in variability of the “bioactivity*, the bioactive/immunoreactive ratio and half-life of different LH isoforms (40). A second issue related to interpretation of LH concentrations is the existence of molecular variants of LH with low immunoreactivity and high bio/immune ratio. The most common is a modification in two amino acids of the LH beta subunit (41), which is very common in Finland but may also be relatively common in other countries. Heterozygotes with this variant (about 24% of the Finnish population) show lower than average LH concentrations in most immunometric assays, and homozygotes (approximately 3% in Finland) will have very low or undetectable LH values (42). This variant appears to have normal bioactivity in adult healthy women (41), although it may be associated with a higher risk of PCOS in some women (43) and slower tempo of puberty in adolescent boys (44). This type of “invisible” or “partially visible” LH may cause puzzling clinical conundrums, with conflicting clinical and laboratory findings which may prompt complex and costly investigations (45), as shown in a vignette from our clinic below (see Box 1).

BOX 1 **Case.**

An 8-year-old boy was referred for early puberty. On PE his height was at the 90th percentile (vs. mid-parental height at the 40th percentile) with evidence of growth acceleration during the last year from his pediatric records, weight at the 60th percentile. Bone age was advanced at 10.5 years. General PE was unremarkable. He had early T3 genital development, with testicular volume of 6–7 ml, pubic hair was stage 2. Laboratory tests showed DHEAS of 30 ug/dl, basal and ACTH-stimulated 17-hydroxyprogesterone <120 ng/dl, total testosterone of 65 ng/dl at 9 AM, FSH of 1.2 IU/L and LH < 0.025 IU/L. A leuprolide stimulation test resulted in a peak FSH of 7.7 IU/L, persistently undetectable LH of <0.025 IU/L at multiple time points, and a 24 h stimulated Testosterone of 260 ng/dl. To exclude a laboratory error, the Leuprolide test was repeated 2 weeks later, with similar results (undetectable baseline and stimulated LH; baseline T of 54 ng/dl, 24 h stimulated testosterone of 233 ng/dl). The 60 min sample was split and sent to 2 different laboratories for the LH assay. While our usual laboratory (Lab A) reported an LH value of <0.025 as mentioned, Lab B reported a value of 5.6 IU/L, which confirmed the clinical diagnosis of CPP, determined to be idiopathic. He was treated with GnRHa with good clinical response and normalization of serum Testosterone to <10 ng/dl. We were unable to obtain genetic studies to evaluate the likely possibility of a variant LH molecule.

**Key Points:** Unlike the old LH RIAs, the current immunometric LH assays are sensitive and specific, allowing detection of early pubertal concentrations in unstimulated LH values in a subset of early pubertal children. However, sensitivity may be suboptimal in “platform” assays used in most hospitals. Conversely, excessive specificity, related to the characteristics of the antibodies used in the assay, or the presence of LH molecular variants with low immunoreactivity, may result in underestimation of LH values.

## Baseline LH measurements for the hormonal confirmation of central precocious puberty (CPP) or delayed puberty

Random LH concentrations are commonly measured in the initial evaluation of children with either premature or delayed pubertal development. Random LH values may be higher in the early morning than at other times during the day (46), although the maximal nocturnal concentrations occur soon after the onset of sleep (33). We and others have shown that random LH concentrations can be used to detect onset of puberty in boys who are “late bloomers”, as LH increases before testicular enlargement is noted (19, 47). The cutoff value considered to be “pubertal” for a random LH value measured with the current, highly sensitive (HS) assays (HS-IFMA, HS ICMA, with limit of quantitation ≤0.05 IU/L), is generally ≥0.3 IU/L (48, 49). The diagnostic sensitivity of a random LH value in the diagnosis of CPP in girls has been evaluated by different groups with variable results, ranging from <50% (50) to 100% (49), with most studies reporting intermediate sensitivity and similarly variable specificity (Table 1). Large studies are more meaningful in this regard. In a large cohort of 449 girls (38), 65% of girls with CPP and 26% of girls with “early normal puberty” (onset between 8 and 9 years) had a random LH > 2 SDS of a control group of girls, and a number of girls with pubertal response to GnRH had prepubertal basal LH values. In another study of over 150 children with CPP, 85% of girls and 97% of boys had a random HS ICMA LH ≥ 0.3 IU/L (48), and the latter subgroup had a generally more advanced pubertal stage than those with lower/undetectable LH. However, some of the girls with stage 4 breast development, and 1 post-menarcheal girl had LH <0.3 IU/L. In another large study (56), the authors noted minimal increase in serum LH concentrations between Tanner B1 and B2, and persistently low LH levels (≤0.2 IU/L) in some children up to stage B4, although this study was marred by using an automated ICMA of suboptimal sensitivity (Immulite 2000 XPi®) which, in our opinion, makes LH values ≤0.3 IU/L difficult to interpret.

**Table 1:** Sensitivity and specificity of basal and stimulated LH values in the diagnosis of puberty.

LH values (IU/L)	Method	Sensitivity (%)	Specificity (%)	Study (reference)	Comments
Unstimulated LH < 0.3 (prepubertal) ≥0.3(pubertal)	ICMA	77–93 48	100 100	Houk et al. (51) Pasternak et al. (52) Neely et al. (49) Logan and Eugster (48) Harrington and Palmert (53) Sathasivam et al. (50)	While a pubertal highly sensitive LH can confirm a diagnosis of CPP, a prepubertal value does not refute it.
Stimulated LH > 4.9 (GnRH)	ICMA	78	79	Pasternak et al. (52)	The transition to a LH-predominant response is often a relatively late development in the clinical progression of central precocious puberty
Stimulated LH > 5 (2 h, Leuprolide)	ICMA	78	100	Sathasivam et al. (50)	
Stimulated LH > 5.5 (3 h, Leuprolide)	ECLIA	93	100	Carretto et al. (54)	
Stimulated LH > 6 (1 h, Triptorelin)	ICMA	89	91	Poomthavorn et al. (55)	

ICMA, immunochemiluminometric assay; ECLIA, electrochemiluminescence; LH, luteinizing hormone; GnRH, gonadotropin-releasing hormone; CPP, central precocious puberty.

The variable diagnostic sensitivity of random LH concentrations at the time of the initial assessment even when measured by highly sensitive assays, is likely related to multiple factors in different studies, in addition to the characteristics of the assay. These include the different intervals between the onset of detectable breast tissue and the hormonal measurements (Tanner B2 may last for 6 or more months), which may be affected by the patterns of referral in different clinics/countries/health care systems, leading to a selection bias; the different time of the day when LH was measured; preselection criteria such as a “pubertal response” to GnRHa (49); and lack of clinical follow-up to document pubertal progression in some studies. Overall, all the quoted and other papers (51, 58) suggest that, while a baseline LH ≥ 0.3 IU/L by highly sensitive IFMA or ICMA is consistent with CPP, a lower random LH value does not exclude CPP. This was clearly stated in a previous document on the use of GnRHa in children (59), and is keeping with a classical observation (based on the LH response to GnRH in the LH-RIA era), that CPP in girls develops along a continuous spectrum of clinical and hormonal changes (60). This concept is reinforced in the above mentioned recent study by Madsen et al. (56) who, by using a sensitive breast ultrasound (US) technique, noted initial breast changes of puberty approximately 2 years before the onset of palpable breast tissue (Tanner B2) and described in detail the continuum of hormonal changes through the pubertal stages. Data are scantier in boys, but it appears that LH values are higher and more frequently in the detectable range in G2 boys than B2 girls (23, 48). A recent large study in boys correlated random gonadotropin and testosterone levels to pubertal stages, assessed both clinically and by testicular sonogram (57).

**Key Points**: random serum LH levels ≥0.3 IU/L by highly sensitive assays suggest the hormonal onset of puberty in both sexes. With less sensitive LH assays, a higher cutoff value may need to be employed (we suggest 0.5 IU/L). It is appropriate to confirm the LH elevation with a repeat, early morning sample, if the purpose is to forgo a GnRH or GnRHa stimulation test. However, undetectable LH concentrations (with any assay) do not exclude the hormonal onset of puberty, and may occur in ∼20%–35% of girls and ∼5% of boys with early hormonal changes that can be detected by a stimulation test.

## Nocturnal LH measurements

The measurement of spontaneous LH secretion by highly sensitive assays in blood samples, collected every 10–30 min could arguably be considered the most physiological method for detection of the early hormonal changes of puberty (31–33, 35, 61, 62). Unfortunately, the procedure is invasive, costly, requires hospitalization, and is generally unavailable outside of a research setting. We will briefly outline below the data regarding correlation of nocturnal LH levels with GnRH and GnRHa-stimulated LH levels.

## Measurement of urinary gonadotropins

Measurement of urinary gonadotropins in timed urine collection by traditional RIA has been available for decades (63, 64), notwithstanding the limited sensitivity of the assay (requiring urine concentration) and reported periodic fluctuations of gonadotropin levels (65). With the advent of the immunometric assays, LH measurement in random or early morning urine samples appeared to provide sufficient sensitivity (66). Urinary gonadotropins increased with advancing age and pubertal development and were detectable in first-morning urinary void, even before physical signs of puberty (67). They were also noted to identify sex-specific gonadotropin changes during early infancy (68). Despite its minimal invasiveness, urinary gonadotropin testing has not become popular in the U.S. However, renewed interest in this procedure has been recently fostered by investigators predominantly from Asian centers (69–71).

**Key Points:** Urinary gonadotropins (LH particularly) provide “integrated” hormonal concentrations and are a useful adjunct for the diagnosis and treatment of CPP. They have been employed mostly in Europe and Asia, and they have not been as extensively studied as the corresponding serum measurements.

## Baseline sex hormones in the diagnosis of puberty

In girls, serum estradiol (E_2_) concentrations are often undetectable or very low (<15 pg/ml or <55 pmol/L) in random daytime blood samples obtained in early to mid-puberty (Breast stage 2–3). In these early pubertal girls, nocturnal pulses of estradiol occur and are sufficient to induce development of secondary sexual characteristics, so that E2 levels can be quantitated, when measured, by a sensitive assay, during the night or in early morning samples. In this regard, it is important to briefly discuss the unextracted, competitive “platform” estradiol immunoassays commonly used in most hospitals. Even though these assays tend to correlate reasonably well (albeit with variable bias) with the reference liquid chromatography-tandem mass spectrometry (LC-TMS) assay at E2 values of ∼25 pg/ml or higher, they correlate poorly with the reference method at lower E2 concentrations (72), thus providing inadequate sensitivity and specificity and misleading results at the low E2 concentrations encountered in early-mid female puberty (73). As mentioned above, the current “gold standard” for serum E2 measurement in the low range seen in (early) pubertal girls is the LC-TMS method (74, 75), with a functional sensitivity of 1–3 pg/ml. Nonetheless, competitive immunoassays which include extraction and column chromatography (76), or even solvent extraction alone (73) provide good sensitivity (∼2–5 pg/ml) and adequate specificity in early female puberty in laboratories that have no access to the more expensive LC/MS/MS method.

In boys, serum testosterone (T) is secreted by the Leydig cells or derives from conversion of adrenal androgens, so that the small T increase (up to ∼30 ng/dl) in early pubertal, adrenarcheal boys cannot be attributed with certainty to gonadal activation. For this reason, measurement of early increases in serum testosterone in boys has lower diagnostic sensitivity than early detection of E2 increase in girls for the diagnosis of CPP, with the understanding that sex hormone measurements should always be paired with serum LH measurements. Thus, serum T measurement by direct, unextracted assays has been used to define diurnal testosterone rhythms in boys and have shown average testosterone concentrations of ∼30 ng/dl in an early morning sample in early puberty, heralded by testicular volume of 3–6 ml (77). Other clinicians have proposed a serum T level of >40 ng/dl to indicate the onset of puberty in boys (78). Nonetheless, the “gold standard” for T measurement in children is the LC-TMS method (57, 79), or an immunoassay involving manual purification steps (80), as direct testosterone assays correlate poorly with the reference method at low T concentrations (81). As an aside consideration, direct testosterone assays are truly inadequate for assessment of hyperandrogenic conditions in pubertal girls or adult women (82–84).

Lastly, high dose (>1–3 mg/day) biotin (vitamin B7) which is available as an over-the counter supplement used to strengthen nails and hair, interferes with the technical aspects of immunoassays and can lead to either falsely elevated or falsely low results when streptavidin binding is utilized in the assay detection system. Biotin does not interfere with LC-MS/MS assays (85).

**Key Points**: E2 levels should be measured by highly sensitive assays (LC-TMS or at least extraction methods) to detect early pubertal changes in girls. Even so, E2 may be truly undetectable or very low at stages B2 and early B3. T values >30–40 ng/dl (measured by a sensitive method, LC-TMS or at least extraction assay) are generally consistent with the onset of hormonal puberty in boys. However, the same levels and especially lower levels above prepubertal (>5–10 ng/dl) may be due to adrenarche, gonadarche or a combination thereof and not be truly diagnostic of gonadarche alone. If immunoassays (i.e., methods not based on mass spectrometry) are used and the results do not make sense clinically, use of high-dose biotin supplements should be excluded.

## Inhibin B levels in the diagnosis of puberty

Inhibin B (INHB) is produced in the Sertoli cells of the testis in males, and in the granulosa cells of the ovary in females. It belongs to the transforming growth factor-β super family and regulates the synthesis and secretion of FSH in a negative feedback loop (86). In males, INHB level reflects Sertoli cell number and function and peaks shortly after birth, decreases during childhood, and then increases at puberty due to FSH stimulation. In females, INHB level is related to the number of antral follicles and reflects the ovarian response to gonadotrophins (87). Undetectable or low INHB levels are observed in boys with either congenital or acquired absence of testicular tissue whereas normal or near-normal levels are seen in cryptorchidism and disorders with preserved Sertoli cell function in spite of absence of germ cells or impaired androgen biosynthesis or action (88). While Individual studies have shown basal inhibin B to have good accuracy to predict the onset of puberty, diagnostic thresholds given by different studies are variable and overlapping. Specifically, there is considerable overlap in INHB concentrations between boys with testicular volume 1–3 ml and those with volume >4 ml (89), thus a single diagnostic cutoff for routine clinical practice is still unavailable (90). Recently, FSH stimulated INHB levels has been explored as a promising investigation for prediction of onset of puberty (91).

**Key Points**: INHB increases at puberty in both sexes, but has been mainly used in males (who have higher values of INHB throughout life), as a marker of puberty. It can be used as an additional hormonal parameter, in the context of a multi-hormonal evaluation, but is inadequate as an isolated maker of puberty in boys.

## Stimulated LH in the diagnosis of puberty

**Figure 1:**
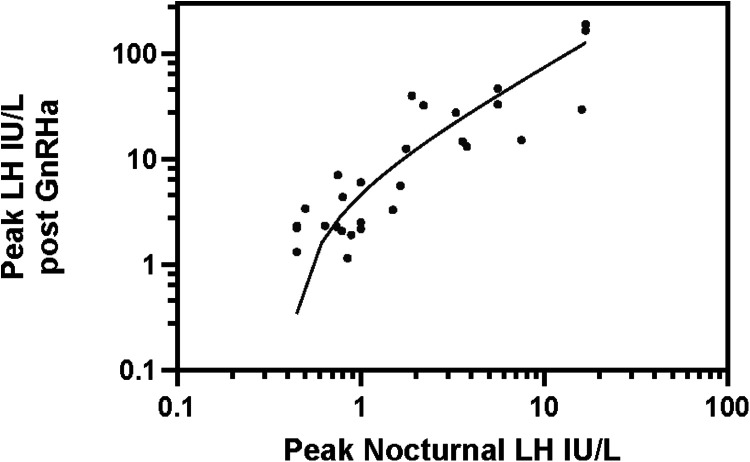
Correlation between nocturnal peak LH vs. Leuprolide stimulated peak LH. y (GnRHa stimulated peak LH) = 7.7 x −3.1 IU/L, where x = peak nocturnal LH. *r* = 0.83; *r*^2^ = 0.69 *p* < 0.001. LH, luteinizing hormone.

In the US, the traditional GnRH stimulation test has been replaced by the GnRHa stimulation test since the mid 1980's, when GnRH became commercially unavailable. Different GnRHa have been used in this country, including parenteral Nafarelin (now unavailable), and, more commonly, subcutaneous (SC) aqueous Leuprolide, which has been used for decades. Triptorelin (which had limited research availability as an aqueous solution in the US) has been used mainly in other countries, and depot preparations of GnRHa have also been used for stimulation, as they release a substantial proportion of the analog rapidly, immediately after injection, resulting in FSH and LH stimulation (92, 93). After an initial study with aqueous SC nafarelin (94), our experience has been limited to aqueous SC leuprolide, which we have been using exclusively for diagnosis of pubertal disorders (95). For monitoring children treated with GnRHa for CPP, we have monitored FSH, LH 1–3 h after stimulation test by aqueous leuprolide and, more recently, by the same Leuprolide depot preparation being used for treatment. We (50, 95) and others (96) have used a Leuprolide dose of 20 mcg/kg. Lower doses of 10 mcg/kg (97) or a single dose of 500 mcg (98) appear to achieve effective stimulation of FSH and LH secretion, but no dose-response studies are available in the 5–20 mcg/kg dose range. Triptorelin has similarly been shown to be effective (55, 99). Although there may be subtle differences in the effectiveness of different GnRHa, probably all of them at the dose commonly employed achieve (sub)maximal FSH and LH stimulation. Few studies have compared the LH response to leuprolide to the response to GnRH stimulation in the same subjects. In a comparison study with testing a few weeks apart, Ibanez (98) showed Leuprolide, 500 mcg SC to induce a LH peak which was almost twice as high as the peak induced by GnRH IV in children with advanced precocious puberty, while response to the 2 agents was similar in prepubertal children. In an analogous, small (*N* = 8), unpublished study of early-mid pubertal girls (Breast T2–T3) with CPP tested with GnRH IV (2.5 mcg/Kg) 3–5 months after undergoing a Leuprolide stimulation test, we found that SC leuprolide (y) achieved a ∼1,4 times higher LH peak average than GnRH despite these patients being a few months older at the GnRH test. These limited data suggest that Leuprolide achieves at least a similar, and possibly greater stimulation of LH (measured by immunometric method) than native GnRH in children evaluated for CPP. Information regarding correlation between the peak LH to Leuprolide and the spontaneous nocturnal LH secretion is likewise limited. Following a study showing good correlation (*r* = 0.83) between nocturnal HS- IFMA LH concentrations and (very) low-dose GnRH-stimulated LH peak values (62), we performed an analogous analysis in 28 early to mid-pubertal girls with premature or early pubertal development. The study group included 25 girls with CPP (progressive on follow-up), and 3 with non-progressive exaggerated thelarche (100) (unpublished data). Of the girls with CPP, 7 [age 6.9 ± 0.5 years (mean ± SD), *Δ*(BA-CA) 1.6 years] were at Tanner stage B2, with LH < 0.25 IU/L in 5, 0.3–0.4 IU/L in 2 girls; baseline (extracted) E2 < 5 pg/ml in 5, 6–7 pg/ml in 2 girls]. Fifteen girls [age 6.8 ± 0.6 years, *Δ*(BA-CA) 2.2 years], were at stage B3, with baseline LH < 0.25 in 4, 0.25–0.5 in 5, 0.5–1.4 IU/L in 6, and E2 < 5 pg/ml in 7, 5–16 pg/ml in 8 girls). Three girls were at stage B4 [8.7 ± 0.3 years, *Δ*(BA-CA) 3.8 years], with baseline LH 1.1–2.7 IU/L, E2 10–25 pg/ml. Three girls with exaggerated thelarche [age 1.7 ± 0.3 years, *Δ*(BA-CA) 1 year] had LH < 0.25 and E2 < 5 pg/ml. In this cohort, we evaluated the relation between 12 h (8 PM to 8 AM) LH-IRMA concentrations (sampled every 30 min) and peak response to Leuprolide (20 mcg/Kg SC). The peak LH (IRMA) after leuprolide showed good correlation with the peak nocturnal LH (*r* = 0.83, *p* < 0.001, Figure 1) and the mean nocturnal LH (*r* = 0.89, *p* < 0.001). A subsequent study from the U. of Chicago, showed that the peak sleep LH concentration, measured during an approximately 3 h period after the onset of sleep, also correlated well (*r* > 0.8) with both the peak LH after Leuprolide, as well as the peak E2 after leuprolide, in normal pubertal girls (101).

While the above information is useful to validate the GnRHa stimulation test and to interpret clinical studies regarding diagnostic sensitivity and specificity of various tests for the diagnosis of CPP, most pediatric endocrinologists have been seeking a specific cutoff value for stimulated LH to diagnose pubertal activation, when baseline LH levels are non-diagnostic. Clinicians have continued to use a cutoff GnRH-stimulated LH value >5 IU/L for the diagnosis of precocious puberty (37), the same value traditionally used LH-RIA, without taking into consideration the differences between RIA and immunometric assays, between different immunometric assays, and the likely potency difference between GnRH and GnRHa (98). Other investigators have proposed various diagnostic threshold values for LH concentrations in the diagnosis of CPP, based on their clinical data. These cutoffs include 3.3 IU/L for IFMA vs. 4.1 for ICMA (102); 6.9 (girls), 9.6 (boys) IU/L for IFMA (39); 8.0 IU/L for ICMA (98); 6.0 IU/L for automated platform chemiluminescent assay (ECLIA) (55); 7 IU/L (IFMA); 8 IU/L (platform ECLIA) (99). Moreover, we have demonstrated that Leuprolide-stimulated LH values by IRMA (95) or HS-ICMA (50) well below the traditional value of 5 IU/L can be associated with CPP. Other investigators have noted that the diagnostic sensitivity of the leuprolide stimulation test, for a stimulated LH cutoff level of 5 IU/L, is highest when the test is prolonged to 180 min, thus allowing the LH to rise maximally (58). In our opinion, defining a universally applicable GnRHa-stimulated LH cutoff value for the diagnosis of CPP is an elusive goal, given the variability and limited standardization of the different LH assays used, the variable data arising from different populations on which proposed threshold values are based, the duration of the GnRHa tests employed, and the fact that, in the continuum of pubertal progression (103), a “low” LH response at one point in time is not necessarily predictive of the speed of future progression. Other caveats regarding interpretation of LH cutoffs to GnRHa include lower values noted in girls with obesity (104). For all these reasons, we think that, for the diagnosis of CPP in girls, the LH response to stimulation, rather than in absolute values, needs to be interpreted in conjunction with the other clinical, radiological and hormonal parameters, including the sex-hormone response to GnRHa stimulation (discussed below), and, as importantly, an observation period of 3–6 months to evaluate progression in cases that are not clear-cut. Because the LH secretion tends to occur earlier in boys than in girls, for the same degree of sexual development in normal and precocious puberty (23, 48), an LH response to GnRHa > 5 IU/L may be theoretically more universally applicable, however data on cutoff LH response to stimulation are limited for boys with CPP, due to the lower prevalence of this condition in males (105–107).

Different cut-points need to be used to interpret random LH concentrations in girls under two years of age because LH concentrations may normally be higher (following the “minipuberty” of infancy); CPP may frequently be misdiagnosed during this phase of development (108). The interpretation of LH response to GnRH or GnRHa is also difficult in these very young girls with premature sexual development as a subgroup of them with idiopathic premature thelarche may show an LH response >5 IU/L to stimulation (108). Additionally, some girls with atypical or “exaggerated” thelarche may show a robust 20–24 h Estradiol response to leuprolide stimulation (100) and still have a self-limited condition.

**Key Points:** The LH response to Leuprolide stimulation correlates with the response to GnRH stimulation and with nocturnal LH secretion (with the limitation that correlation studies are few). Although an LH peak >5 IU/L to stimulation has been considered the hallmark of hormonal puberty, pubertal development in girls can be seen at lower LH values, which may be due to different characteristics among LH assays, the continuum of puberty, or other factors. Thus, a universal “pubertal cutoff” for the LH response to GnRHa may be an elusive goal, at least in girls.

## Sex hormone response to GnRHa in the evaluation of puberty

The original observation that GnRHa administration achieves sequential gonadotropin and estradiol secretion in girls with CPP (94, 95) was subsequently expanded to include an equivalent response in boys, adult men and women (78, 97, 109). Given the above noted difficulties in establishing a diagnostic GnRHa-stimulated LH cutoff value to diagnose precocious puberty in girls, the estradiol response to Leuprolide or other GnRHa (and the analogous testosterone response in boys) can be utilized as evidence of the activation of the entire pituitary-gonadal axis at puberty. We reported that ∼20% of girls with progressive CPP undergoing Leuprolide stimulation did not achieve a predetermined “diagnostic” LH peak value of 5 IU/L by ICMA, yet they could be diagnosed by measuring a peak E2 response >50 pg/ml at 24 h (50). The usefulness of the stimulated (20–24 h post injection) E2 level for the diagnosis of CPP has been supported by other investigators (99, 110) and confirmed in our routine clinical experience (unpublished data), but not all (54). In this regard, the proposed cutoff for peak stimulated E2 value to diagnose CPP ranges from 40 pg/ml (98) to 80 pg/ml (54, 99) and a study suggested that a percentage increase of E2 (>28%) between 3 h and 24 h had the best diagnostic accuracy (110). It is likely that these differences are related both to characteristics of the E2 assay employed, and the variably advanced pubertal development in the different populations studied. For the last few decades, we have found that the ranges we employed in our original report (20–24 h E2 responses to leuprolide > 50 pg ml, by an extracted E2 assay, consistent with progressive/advanced CPP, and responses of 25–50 pg/ml consistent with early/slowly progressive CPP) (95) have correlated well with outcome, follow-up and the need for GnRHa treatment. Nonetheless, we realize that any cutoff value is somewhat arbitrary, also in consideration of the continuum of puberty discussed above for stimulated LH cutoff values.

In boys, there seem to be no consensus about cutoff GnRHa-stimulated T levels for the diagnosis of CPP, as reported series showed baseline T levels in a clear pubertal range in Tanner stage 2 (T2) boys (111), and even in T1 boys (109), thus making the stimulated T values clearly elevated and uninterpretable for diagnostic purposes. Studies of boys with delayed puberty may be more informative in this regard. Three adolescent boys with constitutional delay of puberty (Tanner 1) had an average peak T response of 65 ng/dl, significantly higher than the peak of 20 ng/dl achieved by 8 subjects with hypogonadotropic hypogonadism (112). In a cohort of prepubertal (T1) adolescents and young adults, Lanes et al. showed a peak T response of 29 ± 12 ng/dl in 8 subjects with Gonadotropin deficiency compared to 110 ± 20 ng/dl in 14 subjects with delayed puberty (all subjects were T1), however noted overlap of T responses (as well as LH responses) between the 2 groups (96). In the absence of more definite studies, we have empirically interpreted a cutoff (20–24 h) T response ≥100 ng/dl, or an increment (delta) of >60 ng/dl above baseline, as indicative of CPP in boys, with good clinical correlation. We feel, however, that studies of the testosterone response to GnRHa in normal boys (a larger population than boys with sexual precocity) on the brink of puberty (late T1 stage) would be helpful for interpretation and validation of the cutoff T response to GnRHa for the diagnosis of CPP. While we have found that the LH response to GnRHa is generally sufficient to confirm the diagnosis of CPP in the great majority of boys for their consistent increase in LH secretion at stage T2 (23, 48), the T response can be confirmatory and useful in atypical cases of sexual precocity, as described in the vignette from our clinic (see Box 1).

**Key Points:** As a LH response <5 IU/L (or whatever threshold value is chosen) to GnRHa does not uncommonly occur in girls with CPP, the delayed (20–24 h) E2 response provides an “*in vivo*” bioassay of the activation of the pituitary-ovarian axis. Serum E2 values of 50 pg/ml (range 40–80) or higher (increasing from a low baseline level) are consistent with CPP. Similarly, T values increasing to 100 ng/dl or higher upon GnRHa stimulation in boys are consistent with puberty, although data are limited due to the low incidence of CPP in males.

## Lab evaluation in monitoring GnRHa treatment of central precocious puberty

The efficacy of treatment with GnRHa should be monitored by clinical, radiological and laboratory parameters, as discussed in various review articles (59, 113–115).

Laboratory evaluation should include ultrasensitive LH, FSH and sensitive and specific sex hormone levels (estradiol in girls, testosterone in boys). While clinicians may minimize or forgo blood tests if clinical indices of response to treatment are reassuring, in our center we use at least an initial laboratory assessment of treatment effectiveness approximately 3–5 months after initiation of GnRHa therapy, typically before the 3rd monthly depot-leuprolide injection, before the 2nd injection of a 12 week or 24-week depot preparation, or 2–3 months after placement of the histrelin implant. We suggest subsequent lab monitoring at least yearly, even if clinical evaluation is reassuring. Although the great majority of children respond to the recommended doses of GnRHa, occasional children show inadequate pubertal suppression and may benefit from an increase in the dosage of the GnRHa (for the depot Leuprolide preparations), more frequent administration (if allowed by insurance/healthcare regulations) or switching from an injectable form to a histrelin implant. Clinical trials for the available GnRHas report a range of response in each of the variables (116–122)*.* The reader is referred to several reviews that are available on the different formulations of GnRHa available for treatment of CPP (59, 123–126).

There is disagreement regarding whether a baseline LH level is sufficient, or a GnRHa- stimulated level is preferable to monitor pubertal hormone suppression (117, 127). We have favored the GnRHa-stimulated values, traditionally “the gold standard” (128) which provide a more sensitive measure of LH suppression, although baseline values may be adequate, and “perfect” suppression of the pubertal hormones may not be necessary for a favorable outcome (129). A GnRHa-stimulated LH level <4 IU/L has been used to define biochemical suppression in most studies (59, 117, 126) although we and others (92) have observed lower values (typically <2.5 IU/L) in the great majority of children adequately responding to GnRHa therapy in our Center. As we have noted above, cutoff value variation in different studies can be related in part to the different immunometric LH assays employed. While we have used an LH value at 60 min after aqueous leuprolide stimulation for assessment of pubertal suppression for all patients in the past, we now use an LH measurement 60–90 min after Leuprolide-depot or triptorelin depot, except, of course, in children with the histrelin implant. The release of a large amount of rapidly absorbable GnRHa from the depot preparation has been shown to provide an intense stimulation of gonadotropins (92, 125).

With regard to random LH values, ultrasensitive LH < 0.6 IU/L has been proposed to define biochemical suppression but has not been rigorously studied (127). Others suggest an LH value <1 (130). Again, the cutoff value may be somewhat assay-dependent, which makes it difficult to compare different studies. It is important to note that random highly sensitive LH levels often fail to revert to a prepubertal range even when the pituitary-gonadal axis is fully suppressed (117, 131).

Estradiol and testosterone levels should be low in children adequately responding to GnRHa therapy, provided they are measured by appropriate sensitive and specific assays, as discussed above. In girls, estradiol levels should be <10 pg/ml and are, in fact, often undetectable even when measured by ultrasensitive assays (129). Testosterone values should be <10 ng/dl in pre-adrenarcheal boys, and <20–30 ng/dl in boys with less or more advanced adrenarche. That being said, occasional children (mostly boys in our experience) may have higher sex hormone levels and still show adequate pubertal suppression clinically and by LH monitoring. For this reason, and the fact that sex hormones are often measured by less sensitive and specific unextracted assays, measurement of sex steroids may have lower diagnostic sensitivity than measurement of LH levels.

**Key Points:** For laboratory monitoring of GnRHa therapy, measurement of GnRHa-stimulated LH (±FSH) levels is the “gold standard”, with stimulated LH levels <2.5–4 IU/L indicating adequate suppression of the pituitary-gonadal axis. However, baseline LH < 0.6–1 IU/L may suffice to indicate acceptable suppression. Of note, LH values often remain above prepubertal values (0.3 IU/L) in children adequately treated with GnRHa. With good response to GnRHa therapy, serum E2 levels should be suppressed <10 pg/ml in girls, while in boys T levels <10–20 ng/dl are not always achieved and can still be compatible with adequate suppression. Laboratory monitoring should always be used in conjunction with clinical and radiological evidence of response to therapy. This being said, machine learning algorithms to integrate multiple variables for the diagnosis of CPP are being developed (132).

## Conclusion

Highly sensitive assays are essential to detect the early increase in LH levels at the beginning of puberty. The GnRHa stimulation test activates the entire pituitary-gonadal axis, thus representing a true *in vivo “*bioassay” that quantitates the ability of the child to synthesize sex hormones, the effective markers of pubertal effects on the body. This is particularly relevant for the diagnosis of CPP in girls, as a substantial percentage of them may not achieve a” pubertal” LH (usually considered >5 IU/L, but set at different cutoff values in different studies, as discussed above). Those girls with *bona fide* CPP who do not achieve a “pubertal” LH value on GnRHa stimulation, will most often achieve an E2 peak >50 pg/ml at 20–24 h, indicative of pubertal activation of the pituitary gonadal axis. In boys, measurement of the 20–24 h T response is not as crucial, as most boys with clinical signs of CPP will have stimulated LH responses >5 IU/L, however it may be helpful in corroborating the diagnosis of CPP in atypical cases. Treatment decisions need to be individualized and no one variable alone predicts adequate treatment response.

## Data Availability

The original contributions presented in the study are included in the article/Supplementary Material, further inquiries can be directed to the corresponding author.

## Footnotes

^1^The bioactivity of LH has traditionally been measured by the rat interstitial cell testosterone (RICT)assay described by Dufau et al. (25). In this assay serum from a patient is added to suspension of Leydig cells obtained from rats (or mice in modification of the procedure), and the testosterone released in the medium is quantitated as an index of the LH biopotency.

^2^Functional sensitivity, also referred to as the limit of quantitation, is the minimal concentration at which the intra-assay coefficient of variation (CV) is <20%. The limit of detection, or “sensitivity”, is usually defined as the minimal concentration above 2 SD of the 0 standard.
